# Modeling the Influence of Tool Deflection on Cutting Force and Surface Generation in Micro-Milling

**DOI:** 10.3390/mi8060188

**Published:** 2017-06-17

**Authors:** Dehong Huo, Wanqun Chen, Xiangyu Teng, Chao Lin, Kai Yang

**Affiliations:** 1 The State Key Laboratory of Mechanical Transmission, Chongqing University, Chongqing 400044, China; linchao@cqu.edu.cn; 2 School of Mechanical and Systems Engineering, Newcastle University, Newcastle upon Tyne NE1 7RU, UK; wanqun.chen@newcastle.ac.uk (W.C.); xiangyu.teng@newcastle.ac.uk (X.T.); 3 Center for Precision Engineering, Harbin Institute of Technology, Harbin 150001, China; 4 Army Aviation Institute, Beijing 101123, China; yangkai4545@163.com

**Keywords:** micro-milling, tool deflection, surface generation, cutting force, modeling

## Abstract

In micro-milling, cutting forces generate non-negligible tool deflection, which has a significant influence on the machining process and on workpiece accuracy. This paper investigates the tool deflection during micro-milling and its effect on cutting force and surface generation. The distribution of cutting forces acting on the tool is calculated with a mathematical model that considers tool elasticity and runout, and the tool deflection caused by the cutting forces is then obtained. Furthermore, an improved cutting force model and side wall surface generation model are established, including the tool deflection effect. Both cutting force and surface simulation models were verified by the micro-end-milling experiment, and the results show a very good agreement between the simulation and experiments.

## 1. Introduction

Increasing demands on precision micro-parts, components, and systems have led to the development of micro- and nano-manufacturing technologies. Micro-milling as a newly emerged micro-machining technology is believed to be one of the most versatile and effective micro-machining processes for the fabrication of three-dimensional geometries due to its simple equipment, high processing efficiency, low cost, and low environmental requirements [1,2,3]. In micro-milling operations, the cutting tool diameter is typically between 0.1 and 1 mm. The stiffness of the micro-milling tool is much lower than that of conventional scale tools, so greater tool deflection is expected. Tool deflection caused by cutting forces during machining could affect surface roughness, as well as form and dimensional precision, which is believed to be more prominent in micro-milling compared with its conventional scale counterpart. Therefore, it is imperative that the influence of tool deflections in micro-milling operations be investigated so as to control the cutting process and to predict machining accuracy.

Numerous studies have been carried out on cutting force and surface generation modeling in order to investigate the machining mechanics and dynamics for conventional milling processes. Park and Malekian investigated the cutting force modeling considering the dynamics of the tool, ploughing, and elastic recovery [4]. Afazov et al. studied the cutting force in micro-milling by using the finite element method, and the tool trajectory of and the uncut chip thickness for different parameters were determined and used to predict the cutting forces in micro-milling [5]. Li et al. proposed a new nominal uncut chip thickness algorithm for micro-scale end-milling by considering the combination of an exact trochoidal trajectory of the tool tip and tool run-out [6]. Attanasio et al. investigated the modeling procedure for forecasting cutting forces in micro-channel machining, considering all phenomena involved at the micro-scale [7]. Arizmendi et al. presented a model for predicting surface topography in peripheral milling operations taking tool vibration during the cutting process into account [8]. Ismail et al. presented a surface generation model that showed the effects of tool runout, vibration, and flank wear [9]. Omar et al. proposed a generic and improved model to simultaneously predict the conventional cutting forces along with 3D surface topography during side-milling operations, considering the effects of tool runout, tool deflection, system dynamics, flank face wear, and tool tilting [10]. Yuan et al. proposed a machined surface model of the arbitrary point of the cutter edge with respect to the arbitrary frame of the machining feature point, which can be used for predicting surface topography, surface roughness, and surface location error [11]. Denkena et al. presented a method for the reconstruction of surface topographies of peripheral milled surfaces based on measured cutting forces [12]. Jiang et al. simulated the surface topography considering measured tool displacements in peripheral milling [13]. Lavernhe et al. performed a simulation to study the 3D surface topography of five-axis milling using the N-buffer method [14]. Dépincé and Hascoet predicted surface form errors using a contact point method considering tool deflection caused by the cutting force [15]. Heo et al. estimated the lateral and axial errors of the cutter location caused by the cutting force and compensated by applying an additional feed to the workpiece that is equivalent to the estimated cutter location errors [16]. Rao et al. studied the compensation of surface errors due to cutting force-induced tool deflections in peripheral milling of curved geometries [17]. Mamedov et al. studied the tool deflection in micro-milling considering the stiffness of the milling tool, but the influence of tool deflection on cutting forces was neglected [18]. These previous studies only focused on the tool deflection or surface generation, while the effects of tool deflection introduced by the cutting force on the surface generation were not considered.

Considering the low stiffness of micro-tools used in micro-milling, tool deflection becomes more significant compared with conventional scale milling. Therefore, the influence of tool deflection on surface generation and cutting force must be taken into account during the micro-milling process.

**Figure 1:**
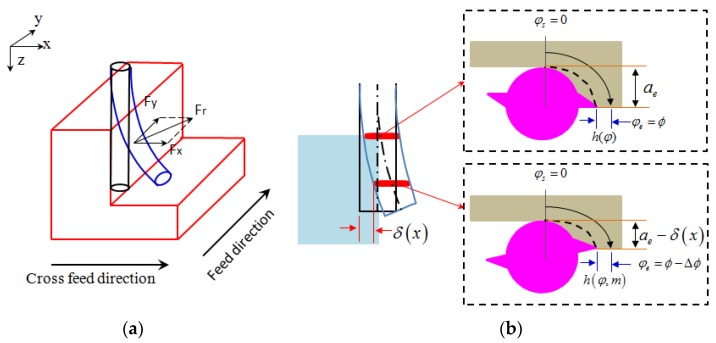
Influence of tool deflection on the radial depth of cut. (**a**) Schematic diagram of tool deflection; (**b**) Top view of the tool deflection

Figure 1a illustrates the effect of tool deflection on the changes in the radial depth of cut and the instantaneous uncut chip thickness. The micro-tool is modeled as a multi cross-section cantilever beam. The top inset of Figure 1b illustrates the entry angle ($\varphi_{s}$) and exit angle $\left(\varphi_{s}\right)$ for a specific radial depth of cut $\left(a_{e}\right)$ and the instantaneous uncut chip thickness $\left(h_{\varphi }\right)$ for a specific feed per tooth, in the ideal cutting state of the milling process without any tool deflection. The bottom inset of Figure 1b illustrates the cutting state when considering the effect of tool deflection. It can be seen that the deflection in the cross-feed direction and the feed direction make the radial immersion and feed per tooth a function of the cutting force and cutting depth, respectively, rather than a specific value as in other cutting force models. It is clear that cutting forces will have a direct influence on the amount of tool deflection, and the tool deflection in turn will form a feedback to the cutting forces. Thus, establishing an accurate cutting force model considering the tool deflection and its feedback effect is important in micro-milling.

In this paper, a surface topography prediction model in peripheral milling taking into account tool deflection is developed, and based on the predicted tool deflection, an improved cutting force model is proposed. Firstly, in order to obtain tool deflection of the cutter, the initial cutting force calculated with a known simulation model is thrust upon the cutter. The tool deflection is then used to update the cutting force. After numerous iterations, the final tool deflection is obtained and used to predict the surface topography. Finally, the models are validated by micro-milling experiments through comparison of the surface topography and cutting force.

## 2. Surface Generation and Cutting Force Prediction

In this section, the influence of the tool deflection on the surface generation and cutting force is presented. The proposed model is developed for both up- and down-milling. In the model, the workpiece is assumed to be rigid. 

**Figure 2:**
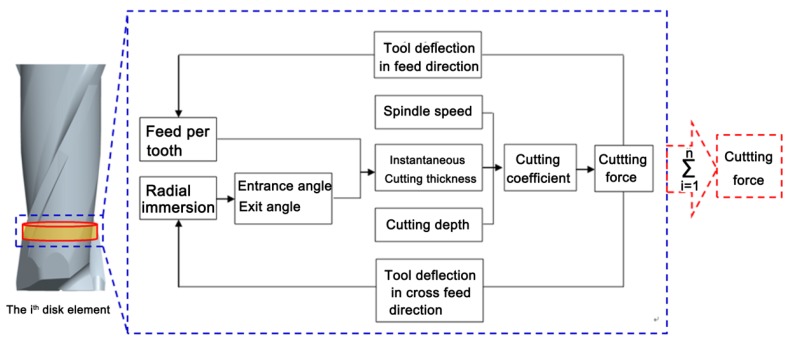
Flow chart of the improved cutting force model.

Figure 2 illustrates the flow chart of the proposed cutting force model, firstly, the cutter is discretized into elements along the cutting depth, and the initial cutting forces for each element are calculated according to the machining parameters including feed per tooth, radial immersion, and spindle speed. Then, the tool deflection in both the feed direction and the cross-feed direction caused by the cutting forces are predicted, and the influence of the tool deflection on feed per tooth and radial immersion are obtained to update the new cutting force. After numerous iterations, the final tool deflection and the cutting force are acquired.

### 2.1. Cutting Force Calculation

**Figure 3:**
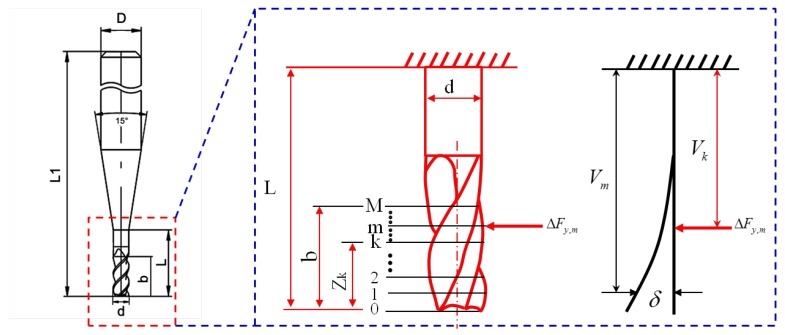
Schematic of tool deflection calculation algorithm.

In order to calculate the cutting forces due to deflection, the micro-tool is simplified as an elastic cantilever round beam that provides the same 2nd moment of area as the actual micro-milling tool. Through cross-section area measurement of the tool used in this paper, the diameter of the simplified round beam is determined as 80% of the actual micro-tool diameter, D. As shown in Figure 3, the tool in the cutting depth is discretized into small differential elements with height *dz*, each element is noted as *m* (*m =* 0, 1, *…*, *M*).

(1) $$M=\frac{b}{dz}$$

where *b* is the cutting depth.

The tool deflection caused by the cutting force acted on element *m* can be given as

(2) $$\delta_{y}\left(z_{k,m}\right)=\{\begin{array}{c} \left(3\upsilon_{m}-\upsilon_{k}\right)\frac{\Delta F_{y,m}\upsilon_{m}^{2}}{6EI}\;0<\upsilon_{k}<\upsilon_{m}\\ \left(3\upsilon_{k}-\upsilon_{mk}\right)\frac{\Delta F_{y,m}\upsilon_{m}^{2}}{6EI}\;\upsilon_{m}<\upsilon_{mk} \end{array}$$

where *E* denotes Young’s modulus of the cutting tool material, *I* denotes the 2nd moment of area, $∆F_{y,m}$ denotes the cutting force in the *y*-direction of the element *m*, $Z_{k}$ denotes the distance from the end of the tool to the element *k*, *Z_m_* denotes the distance from the end of the tool to the element *m*, $V_{k}$ denotes the distance from the fixed end of the tool to $∆F_{y,m},\;\;V_{m}$ denotes the distance from the fixed end of the tool to the element *m*. $I=\frac{\pi {\left(0.8D\right)}^{4}}{64}$, $\upsilon_{k}=l-Z_{k}$, and $\upsilon_{m}=l-Z_{m}$. Substituting *y* with *x*, the tool deflection in the *x*-direction can be obtained.

The cutting forces for element *m* in the *x-* and *y*-directions are

(3) $$\{\begin{array}{c} ∆F_{x,m}\left(\varphi \right)=h\left(\varphi_{j}\right)∆z\sum_{j=0}^{N-1}\left[K_{t}cos\varphi_{j}\left(z\right)+K_{r}sin\varphi_{j}\left(z\right)\right]g_{m}\left(\varphi_{j}\right)\\ ∆F_{y,m}\left(\varphi \right)=h\left(\varphi_{j}\right)∆z\sum_{j=0}^{N-1}\left[K_{t}sin\varphi_{j}\left(z\right)-K_{r}cos\varphi_{j}\left(z\right)\right]g_{m}\left(\varphi_{j}\right) \end{array}$$

where *K_t_* and *K_r_* denote the cutting force coefficients, and

(4) $$g_{m}\left(\varphi_{j}\right)=\{\begin{array}{c} \begin{array}{cc} 1 & \varphi_{e,m}\leq \varphi_{j}\leq \varphi_{s,m} \end{array}\\ \begin{array}{cc} 0\; & other\; \end{array} \end{array}$$

The entry angle for up-milling is $\varphi_{s}=0$, while the exit angle, $\varphi_{e}$, depends on the radial depth of cut, *a_e_*, and the tool radius, *r*. Without tool deflection, the radial depth of cut is a constant, so the exit angle is expressed by

(5) $$\varphi_{e}=cos^{-1}\left(\frac{r-a_{e}}{r}\right)$$

In down-milling, the exit angle is $\varphi_{e}=180{}^{\circ}$, the entry angle is expressed by

(6) $$\varphi_{s}=180-cos^{-1}\left(\frac{r-a_{e}}{r}\right)$$

It was found that the entry and exit angles are kept the same for each element when tool deflection is not considered.

For micro-milling, the feed per tooth to tool radius (*f_t_*/*r*) ratio is usually greater than 0.1, so the circular tool path approximation will introduce larger errors. Moreover, the tool runout, *r*_0_, to tool diameter ratio, (*r*_0_/*r*) becomes larger, so the runout has a significant influence on the cutting force. In order to considering the runout effect on micro-milling, Bao and Tansel [19] established an analytical cutting force model for micro-end-milling with a tool runout effect. The trajectory of the tool tip was given as

(7) $$\{\begin{array}{c} x_{1}=f_{t}zn+rsin\left[\omega t-\frac{2\pi \left(z_{i}-1\right)}{Z}\right]+r_{0}sin(\omega t+\gamma )\\ y_{1}=rcos\left[\omega t-\frac{2\pi \left(z_{i}-1\right)}{Z}\right]+r_{0}cos\left(f_{y}t+\gamma \right) \end{array}$$

and the chip thickness was given as

(8) $$h\left(\varphi \right)\approx f_{t}\left[1+{\left(-1\right)}^{z}\frac{2r_{0}}{\pi r}sin\gamma \right]sin\varphi -\frac{1}{\pi r}f_{t}^{2}sin\varphi cos\varphi +\frac{1}{2r}f_{t}^{2}{cos}^{2}\varphi -{\left(-1\right)}^{z}2r_{0}cos\gamma$$

where *z* is the number of the tooth, *f_t_* is the feed per tooth, *n* is the spindle rotation speed and γ is the runout angle.

Considering the deflection in feed direction, the instantaneous cutting thickness of element *m* is

(9) $$\begin{array}{l} h\left(\varphi ,m\right)\approx \left(f_{t}-\delta_{x}\left(z_{k},m\right)\right)\left[1+{\left(-1\right)}^{z}\frac{2r_{0}}{\pi r}sin\gamma \right]sin\varphi -\frac{1}{\pi r}{\left[f_{t}-\delta_{x}\left(z_{k},m\right)\right]}^{2}sin\varphi cos\varphi \\ +\frac{1}{2r}{\left[f_{t}-\delta_{x}\left(z_{k},m\right)\right]}^{2}{cos}^{2}\varphi -{\left(-1\right)}^{z}2r_{0}cos\gamma \end{array}.$$

Considering the tool deflection effect in the cross-feed direction, the exit angle for element *m* in up-milling becomes

(10) $$\varphi_{e,m}=cos^{-1}\left(\frac{r-a_{e}+\delta_{y}\left(z_{k,m}\right)}{r}\right)$$

Similarly, in down-milling, the entry angle becomes

(11) $$\varphi_{s,m}=180-cos^{-1}\left(\frac{r-a_{e}+\delta_{y}\left(z_{k,m}\right)}{r}\right)$$

It can be noted that, when considering the tool deflection, each slice has a different entry and exit angle, the value of $g_{m}\left(\varphi_{j}\right)$ changes for each element rather than a constant value, so the total cutting force varies significantly.

The total cutting force in the *x-* and *y*-directions can be obtained as

(12) $$\{\begin{array}{c} F_{x}=\sum_{i=1}^{Z}\sum_{j=1}^{M}h\left(\varphi_{j}\right)∆z\left[K_{t}cos\varphi_{j}\left(z\right)+K_{r}sin\varphi_{j}\left(z\right)\right]g_{m}\left(\varphi_{j}\right)\\ F_{y}=\sum_{i=1}^{Z}\sum_{j=1}^{M}h\left(\varphi_{j}\right)∆z\left[K_{t}sin\varphi_{j}\left(z\right)-K_{r}cos\varphi_{j}\left(z\right)\right]g_{m}\left(\varphi_{j}\right) \end{array}$$

### 2.2. Side Wall Surface Generation Model 

**Figure 4:**
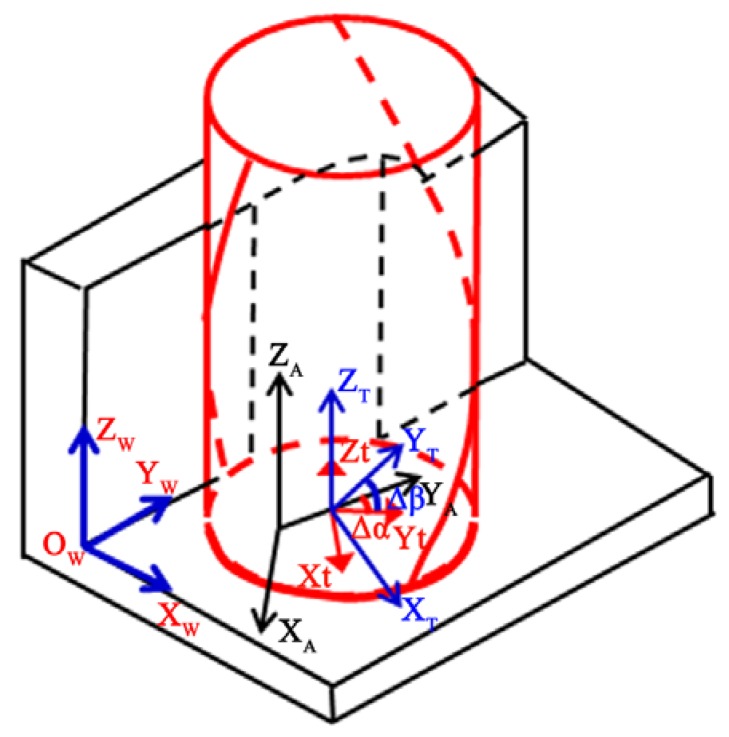
Definition of reference coordinates system for surface generation modeling.

The side wall surface topography in the end-milling process is mainly formed by the cutting paths of the main cutting edges. The main cutting edge of the end mill is a spatial curve for the cylindrical helix, as shown in Figure 4.

In Figure 4, four coordinate systems are defined for surface generation modeling in side wall milling and their transformation matrix used in the model are explained below. 

*O_t_*-*X_t_Y_t_Z_t_* is defined as the local coordinate system of the tool, which considers the rotation of the tool around the spindle axis and the translation relative to the workpiece. The tool center is set as the origin of the coordinate system, the *Z_t_* axis is set to align with the tool axis, assuming that the diameter of the cutter is *r*, the helix angle is β, and the equation of the main cutting edge can be expressed as

(13) $$\{\begin{array}{c} x_{t}=rcos\alpha \\ y_{t}=rsin\alpha \\ Z_{t}=r\alpha cot\beta \end{array}$$

where *α* is the rotation angle.

The matrix expression of the cutting edge coordinates in *O_t_*-*X_t_Y_t_Z_t_* is given as

(14) $$\left[\begin{array}{c} x_{t}\\ y_{t}\\ \begin{array}{c} z_{t}\\ 1 \end{array} \end{array}\right]=\left[\begin{array}{c} rcos\alpha \\ rsin\alpha \\ \begin{array}{c} r\alpha cot\beta \\ 1 \end{array} \end{array}\right]$$

In order to simplify the calculation process, the equation of the main cutting edge can be expressed by

(15) $$\left[\begin{array}{c} x_{t}\\ y_{t}\\ \begin{array}{c} z_{t}\\ 1 \end{array} \end{array}\right]=\left[\begin{array}{c} rcos\left(\frac{z}{rcot\beta }\right)\\ rsin\left(\frac{z}{rcot\beta }\right)\\ \begin{array}{c} z\\ 1 \end{array} \end{array}\right]$$

*O_T_*-*X_T_Y_T_Z_T_* is defined as the coordinate system of the tool, which only considers the translation relative to the workpiece. The angle between *X_T_* and *X_t_* is ∆α, and axis *Z_T_* is parallel to *Z_t_*. The angular velocity of *O_t_*-*X_t_Y_t_Z_t_* relative to the *O_T_*-*X_T_Y_T_Z_T_* is the angular velocity of the spindle. Since the relative rotational angle, *θ*, of the two coordinate systems changes with time, when the points of the cutting edge changing to *O_T_*-*X_T_Y_T_Z_T_*, the rotational effect is considered.

*O_A_*-*X_A_Y_A_Z_A_* is defined as the spindle coordinate system, which describes the relative translation of the spindle and workpiece. The angle between *X_A_* and *X_t_* is ∆β, and axis *Z_A_* is parallel to *Z_t_*. By transforming *O_T_*-*X_T_Y_T_Z_T_* to *O_A_*-*X_A_Y_A_Z_A_*, the runout of the spindle in axial and radial directions is considered. The amplitude and initial phase angle of radial runout is Δd and Δα, and the amplitude and initial phase angle of axial runout is Δh and Δβ, respectively.

*O_W_*-*X_W_Y_W_Z_W_* is defined as the coordinate system of the workpiece, *Z_W_* is parallel to *Zt*. There is only relative movement between the *O_W_*-*X_W_Y_W_Z_W_* and *O_A_*-*X_A_Y_A_Z_A_*, and the initial relative position of the origin of the *O_W_*-*X_W_Y_W_Z_W_* and the origin of the *O_A_*-*X_A_Y_A_Z_A_* is determined according to the actual machining situation, which can be expressed as

(16) $$\{\begin{array}{c} x_{0A}=x_{0}\\ y_{0A}=y_{0}+v_{f}t\\ z_{0A}=z_{0}+\left(i-1\right)a_{p} \end{array}$$

where $x_{0},y_{0},\;\mathrm{and}\;z_{0}$ denote the initial coordinate of the workpiece, $v_{f}$ denotes the feed speed, and $a_{p}$ denotes the cutting depth of one cutting path.

Thus, by transforming the coordinates of the tool cutting edge in *O_t_*-*X_t_Y_t_Z_t_* into *O_W_*-*X_W_Y_W_Z_W_*, the cutting edge equation in the workpiece coordinate system can be expressed as

(17) $$\left[\begin{array}{c} x_{\omega }\\ y_{\omega }\\ \begin{array}{c} z_{\omega }\\ 1 \end{array} \end{array}\right]=T\left[\begin{array}{c} x_{t}\\ y_{t}\\ \begin{array}{c} z_{t}\\ 1 \end{array} \end{array}\right]=\left[\begin{array}{c} Rcos\left(\theta_{i}+\alpha \right)+∆dcos\left(∆\alpha -\omega t\right)+x_{0}\\ Rsin\left(\theta_{i}+\alpha \right)+∆dsin\left(∆\alpha -\omega t\right)+y_{0}+v_{f}t\\ \begin{array}{c} z+∆hsin\left(∆\beta -\omega t\right)+z_{0}+\left(i-1\right)a_{p}\\ 1 \end{array} \end{array}\right]$$

where *T* is the homogeneous transformation matrix from *O_t_*-*X_t_Y_t_Z_t_* to *O_W_-X_W_Y_W_Z_W_*.

By considering each point on the cutting tooth, the tool trajectory is updated in a specific time interval. The cutter profile sweeps along the updated tool trajectory to generate the actual cutting path by using Equation (17). Then, the *Z* map technology is used to plot the minimum *z* against *x* and *y* in the workpiece coordinate system. Thus, the final machined surface can be generated.

## 3. Simulation and Experimental Results

**Figure 5:**
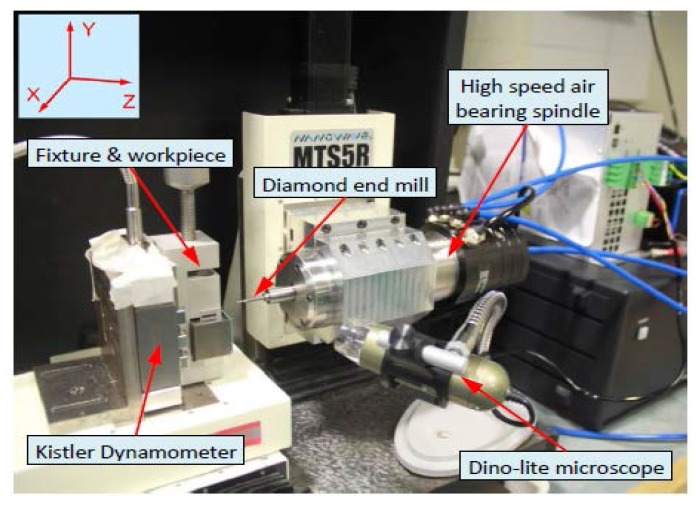
Machining system setup.

The machining experiments were performed on a three-axis precision milling machine tool (Nanowave MTS5R, Nano Corporation, Yokohama, Japan). The machine tool was equipped with three precision linear stages, which are driven by DC servo motors with the smallest feed of 0.1 µm, and a high speed of spindle (5000–80,000 rpm). A typical experimental setup is presented in Figure 5. A three-component piezoelectric dynamometer (Kistler 9256C2, Kistler Instruments Ltd., Hampshire, UK) is mounted on the *X*–*Z* stages to measure feed and cross-feed cutting forces. Aluminum 7075 workpiece was clamped on a fixture attached to the dynamometer. Uncoated tungsten carbide square end mills with diameter of 0.5 mm were used. Identical machining parameters were used in both simualtion and machining experiments: an axial depth of cut of 0.05 mm, a radial depth of cut of 0.3 mm, a spindle speed of 30,000 rpm, and a feed per tooth of 15 μm.

**Figure 6:**
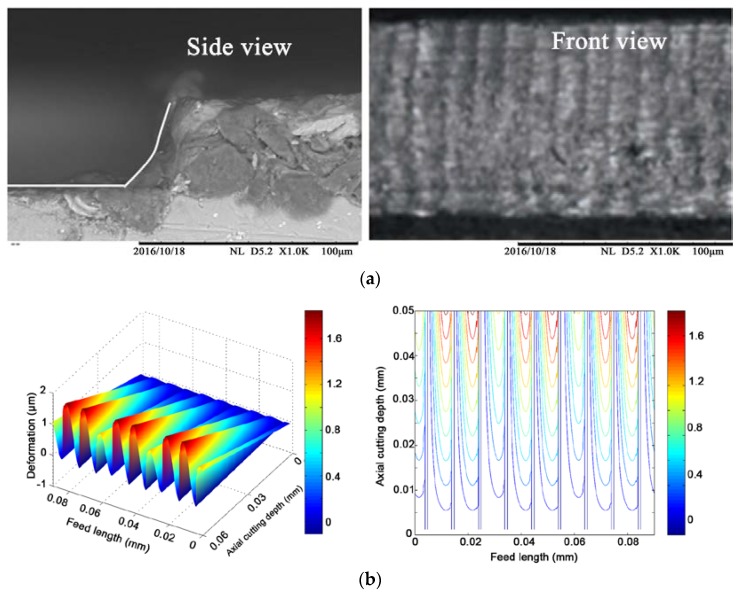
Simulated and micro-milled side wall surface under the same cutting parameters. (**a**) Test results of SEM; (**b**) Simulation results.

Figure 6a shows the scanning electron microscope (TM3030, Hitachi High-technologies Corporation, Tokyo, Japan) images of the machined side wall surface. From the side view of the workpiece, it can be noted that the side wall profile of the slot is a curve due to tool deflection rather than a straight line. The front view of the machined side wall surface shows that a wavy surface was generated, and the amplitude of the wave increases along the cutting depth direction. Figure 6b shows the simulated side wall surface using the proposed model. A good agreement in surface topography and wave magnitude was obtained, which verifies the proposed simulation model.

**Figure 7:**
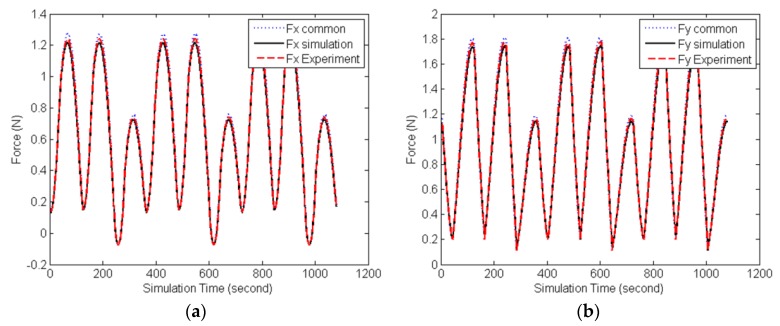
Simulation and experiment cutting forces. (**a**) Cutting force in *x* direction; (**b**) Cutting force in *y* direction.

Figure 7 shows a comparison between simulation and experiment cutting forces in the *x-* and *y-*directions, respectively. It was found that the cutting force predicted by common cutting force simulation model without considering the tool deflection is larger than the experiment cutting force, while in the proposed simulation model considering the deflection of the tool caused by the cutting force, the cutting force can be predicted more accurately.

## 4. Conclusions

In this paper, the influence of the tool deflection during micro-milling on the cutting force and surface generation are investigated, and an improved cutting force prediction method is proposed considering tool deflection. The actual cutting force is found to be smaller than the ideal cutting force because of the tool deflection. Tool deflection in feed and cross-feed directions affects the feed per tooth and the radial immersion in the machining process, respectively, which cannot be neglected in precision micro-milling. These factors were taken into account for the cutting force calculation and were used to update the cutting parameters so that an accurate surface topography generation can be predicted. The comparison between the prediction and the experimental results shows very good agreement.
